# Supplementary material for: Spin-ice physics in cadmium cyanide
Source: Nat Commun. 2021 Apr 15;12:2272. doi: 10.1038/s41467-021-22515-3 (PMC8050284; doi:10.1038/s41467-021-22515-3)
Supplement: Supplementary file 1 — Supplementary Information [file 41467_2021_22515_MOESM1_ESM.pdf]

SUPPLEMENTARY FIGURES

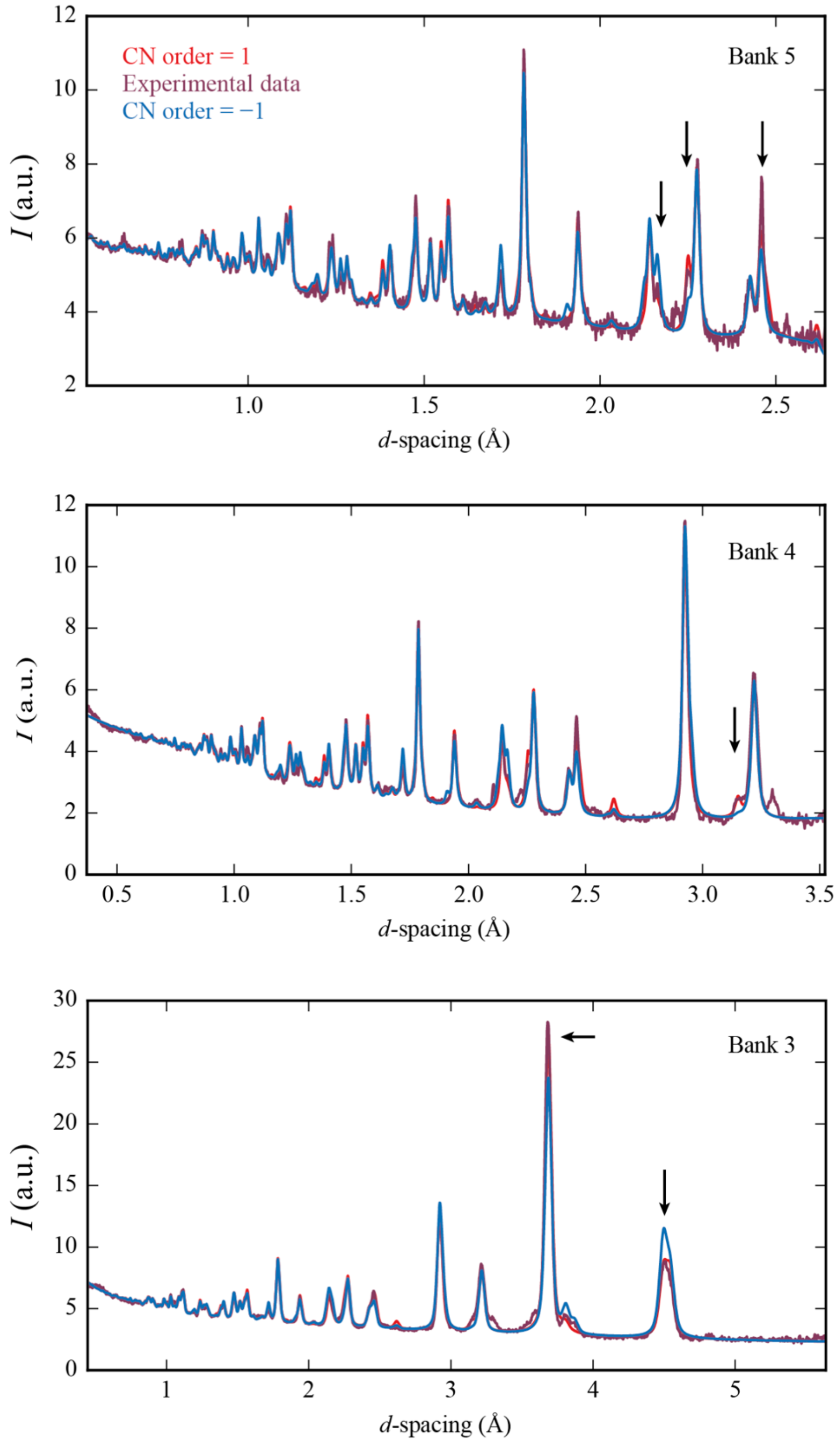

Supplementary Figure 1. **Sensitivity of neutron diffraction to CN orientations.** Calculated and observed neutron diffraction patterns for $Cd(CN)_2$ at 10 K, measured at three detector banks on the Polaris instrument. In each case we compare the experimental data (purple) with diffraction profiles calculated for fully-orientationally-ordered $I4_1/amd$ $Cd(CN)_2$ models (red and blue lines for opposing senses of CN^- order). The peaks which show particular sensitivity to cyanide order are indicated with arrows. The cyanide orientational order parameter determined by Rietveld refinement for this data set is 0.43(3).

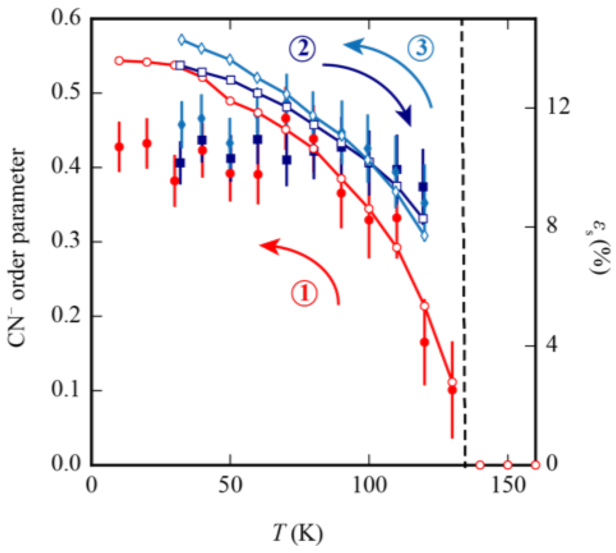

Supplementary Figure 2. **Evidence of kinetic effects in $Cd(CN)_2$ cyanide ordering.** Cyanide order parameter (filled markers) and spontaneous strain values (open markers) for three different heating/cooling cycles; error bars denote the standard errors obtained from Rietveld refinement and, for the strain, are smaller than the symbols. The data measured on cooling from room temperature, corresponding to those in the main text, are shown as red circles. In an additional experiment, the sample was quench cooled from room temperature to 30 K and held at 30 K for a period of one month before heating to 125 K (dark blue squares), then re-cooled to 30 K (light-blue diamonds). Evidently the rate and direction of cooling/heating affects both the lattice parameters and degree of cyanide order.

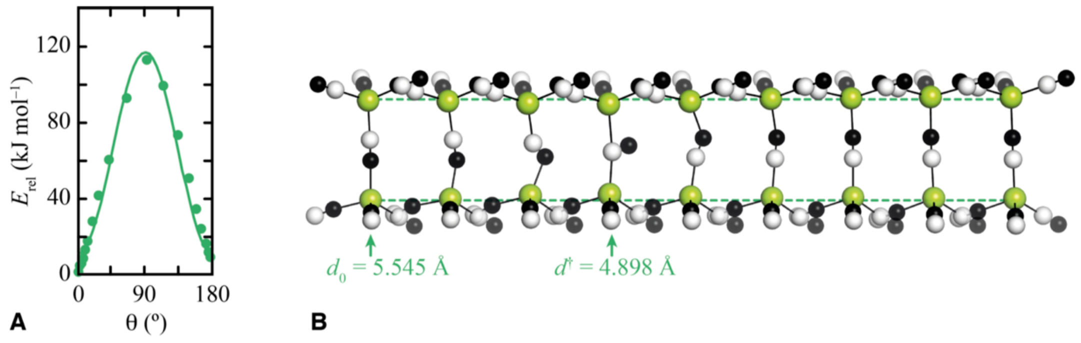

Supplementary Figure 3. **CN flip pathway from nudged elastic band calculations. a** Representative nudged elastic band calculation energies (PBE-D3 functional) for a 180° CN^- orientation flip (filled green symbols) and corresponding fit $E(\theta) = \Delta\cos^2\theta = \Delta S_{\parallel}^2$ (solid green line), from which the value of Δ was obtained. **b** Representative intermediate structures for a single cyanide reorientation. The Cd…Cd distance is reduced during cyanide flipping. The dashed green line acts as a guide to the eye, indicating the equilibrium Cd…Cd distance.

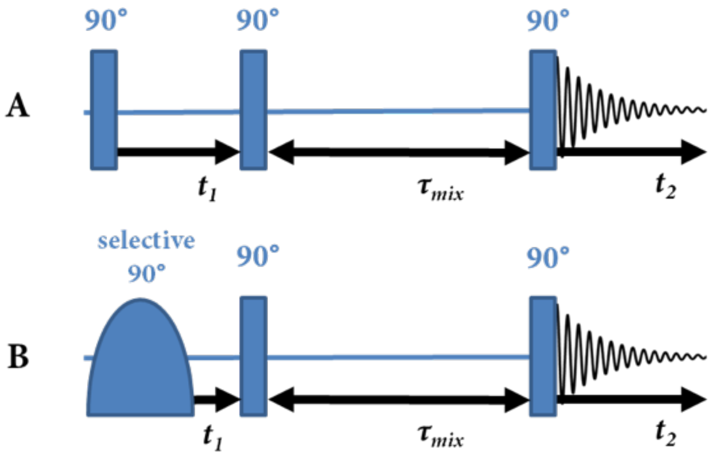

Supplementary Figure 4. **Pulse sequences used for exchange spectroscopy. a** Conventional 2D sequence to visualise slow motion during the mixing period. **b** 1D variant for selective excitation of one resonance before mixing.

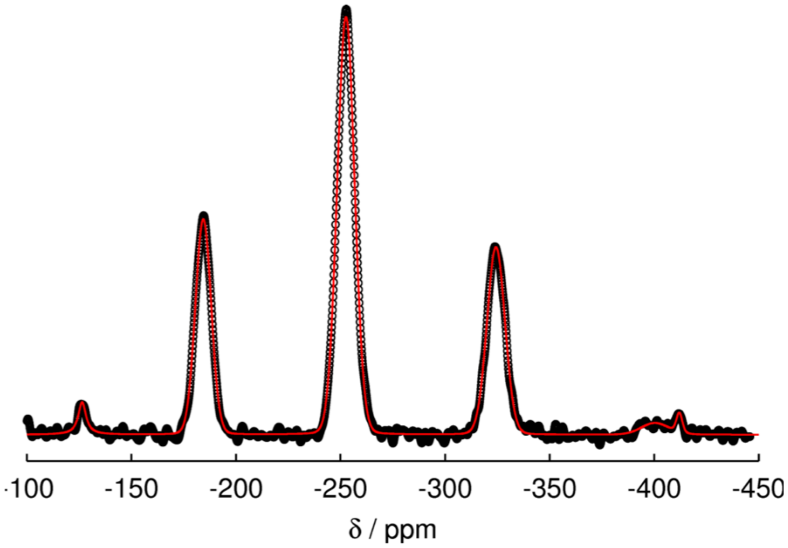

Supplementary Figure 5. **Representative MAS spectrum of $Cd(CN)_2$.** ^{113}Cd MAS spectrum at room temperature acquired with a spinning speed of 20 kHz. Experimental data are depicted as black circles and the deconvolution is shown as a red line. The spectrum consists of five resonances representative of the five possible cadmium environments.

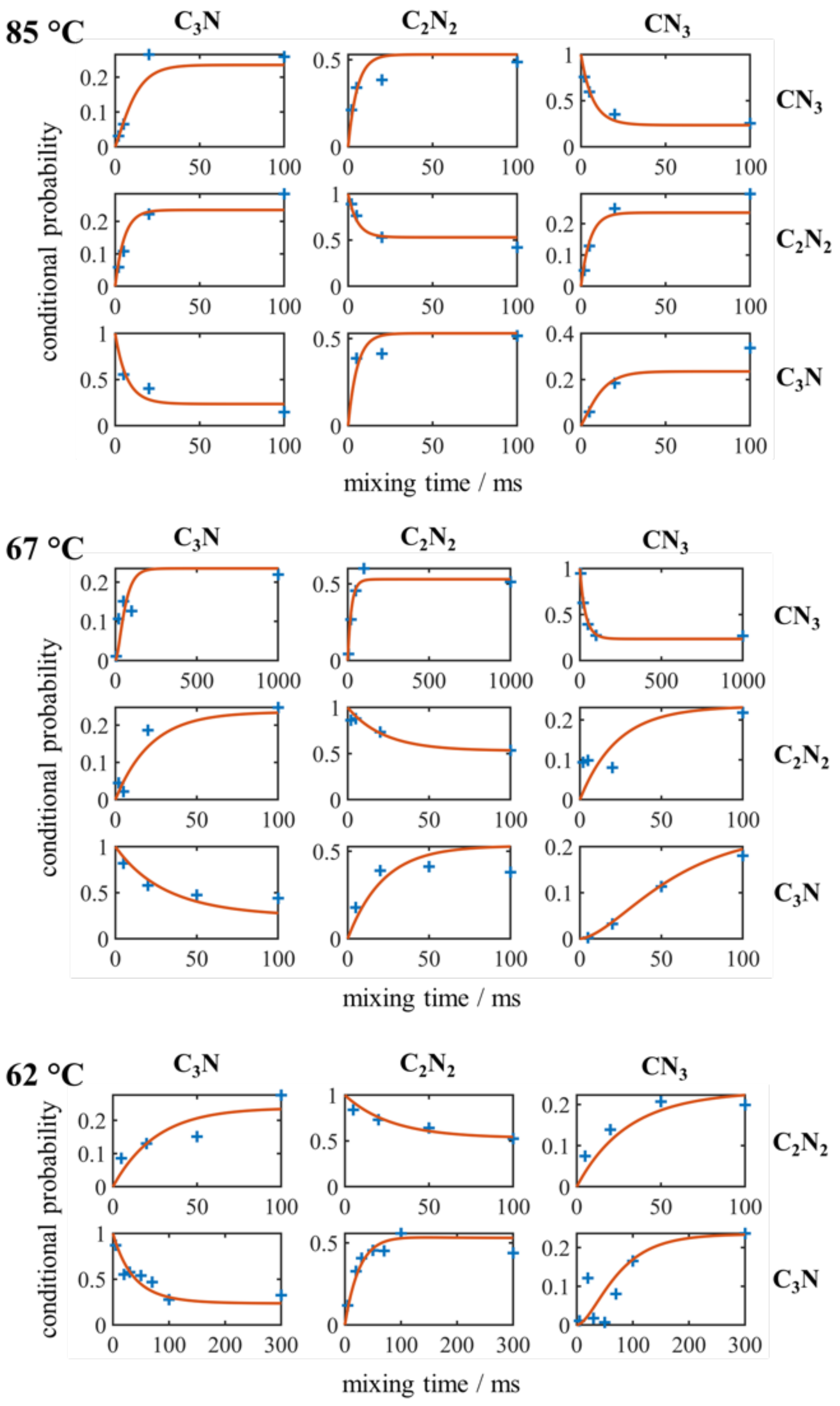

Supplementary Figure 6. **CN flip dynamics from EXSY measurements.** ^{113}Cd conditional probabilities depending on EXSY mixing time for exchange between $CdC_{4-x}N_x$ species for 85 °C (top), 67 °C (center), and 62 °C (bottom). Each row corresponds to one selectively excited resonance before the mixing interval, while each column represents the buildup of the corresponding resonance after the mixing time. Measured data are indicated by blue crosses (+), and fits based on the rate matrix approach are depicted by straight lines.

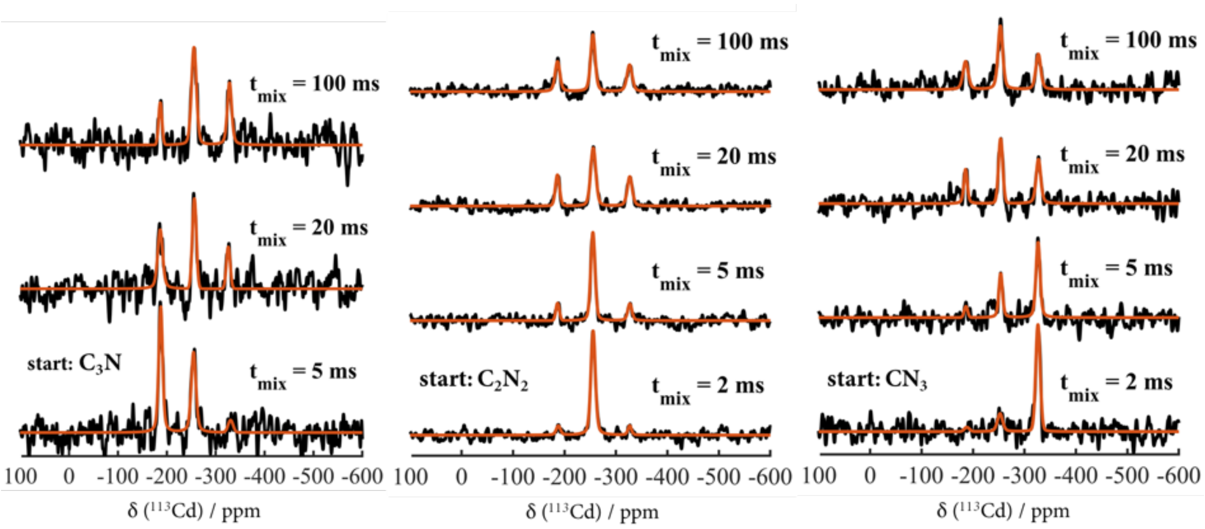

Supplementary Figure 7. **Representative EXSY measurements at 85 °C.** ^{113}Cd selective 1D EXSY measurements at 85 °C (black) with selective excitation of one specific resonance (denoted “start”) per stack. Fits of the $CdC_{4-x}N_x$ resonances are shown in red.

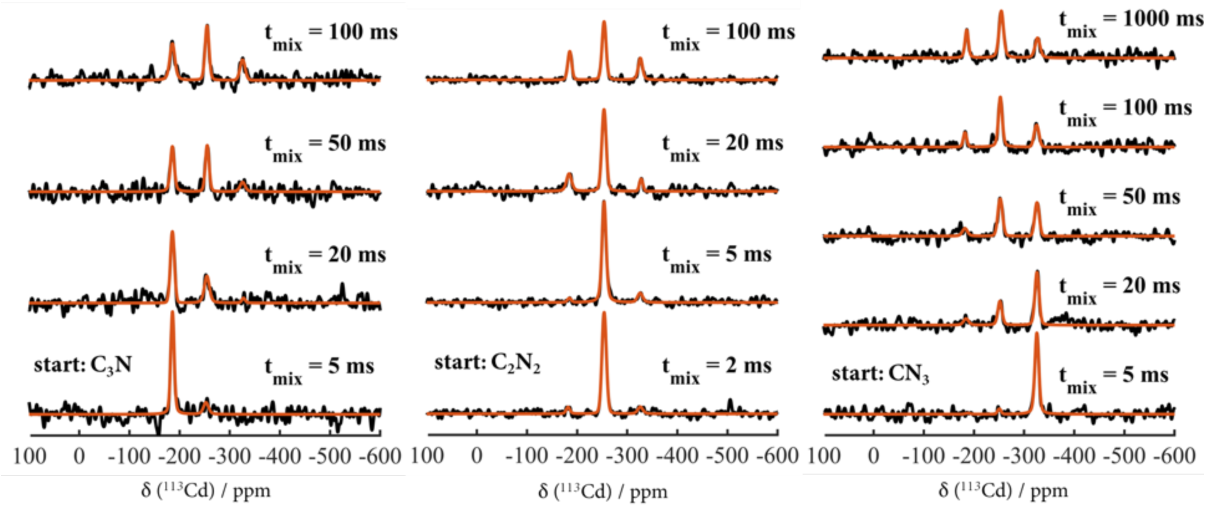

Supplementary Figure 8. **Representative EXSY measurements at 67 °C.** ^{113}Cd selective 1D EXSY measurements at 67 °C (black) with selective excitation of one specific resonance (denoted “start”) per stack. Fits of the $CdC_{4-x}N_x$ resonances are shown in red.

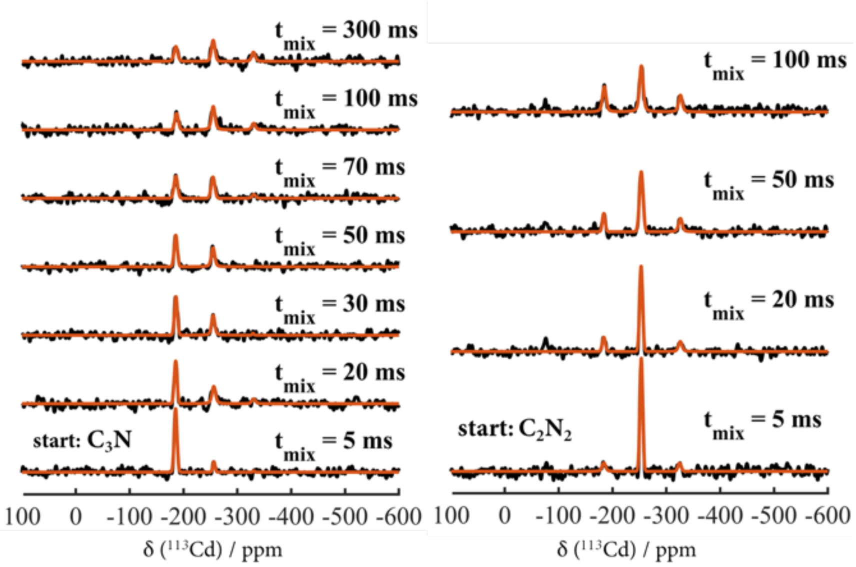

Supplementary Figure 9. **Representative EXSY measurements at 62 °C.** ^{113}Cd selective 1D EXSY measurements at 62 °C (black) with selective excitation of one specific resonance (denoted "start") per stack. Fits of the $CdC_{4-x}N_x$ resonances are shown in red.

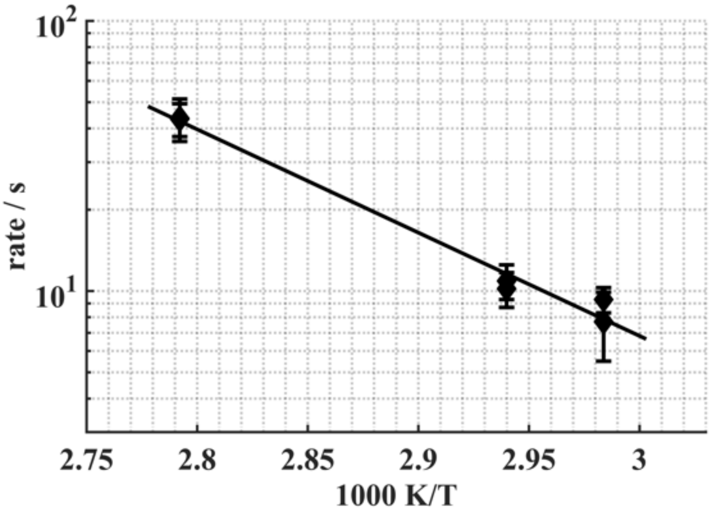

Supplementary Figure 10. **Determination of the CN flip barrier height.** Arrhenius plot of the exchange rates from CdC_2N_2 to CdC_3N (k_{12}) and from CdC_2N_2 to $CdCN_3$ (k_{32}): the fitted activation energy of the CN^- flip is 73 (5) kJ mol^{-1}.

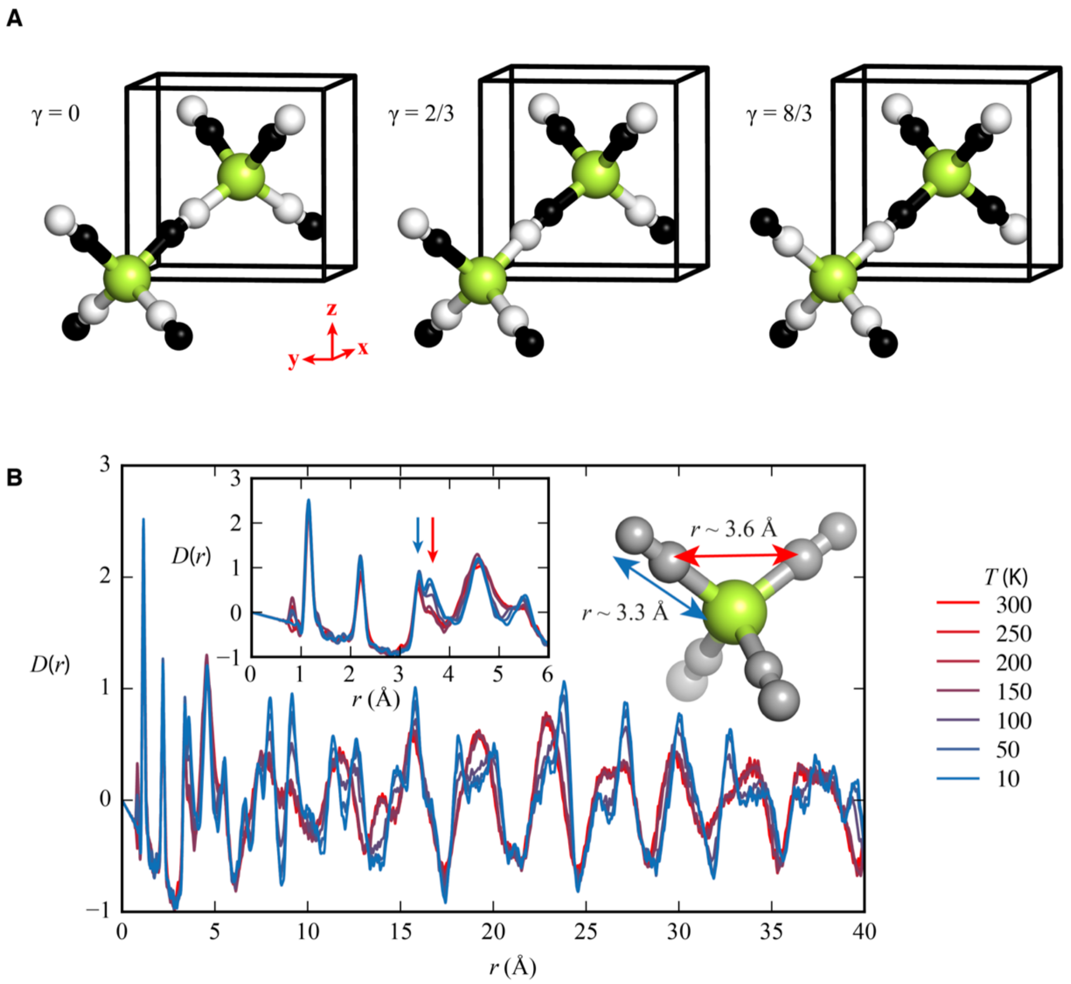

Supplementary Figure 11. **Neutron PDFs of $Cd(CN)_2$ and their analysis. a** Structures of $Cd(CN)_2$ obtained from *ab initio* calculations for relaxations of single cells with different cyanide decorations. Cd in green, C in white and N in black. These structures were used to calculate $D(r,\gamma = 0)$ and $D(r, \gamma = \frac{2}{3})$ used in our PDF analysis to determine the relative populations of coordination environments as a function of temperature. **b** PDF data collected on cooling between T = 300 and 10 K. The peaks in the range $3.1 < r < 3.8$ Å are most directly sensitive to the local CdC_xN_{4-x} decoration. The approximate distances for specific interatomic correlations are indicated for the disordered average structure. Disordered C/N shown in grey.

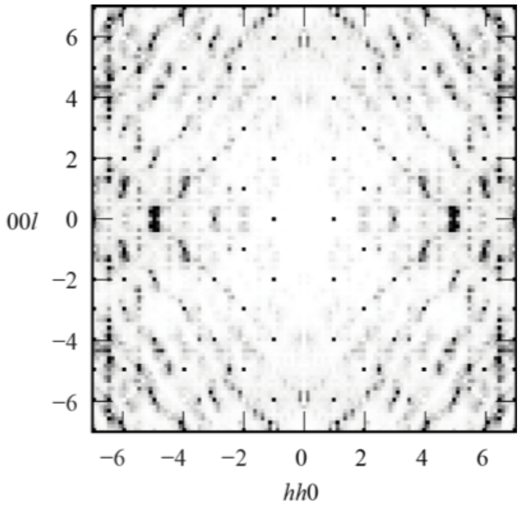

Supplementary Figure 12. **Single-crystal neutron diffuse scattering.** (*hhl*) plane of calculated single-crystal neutron diffuse scattering based on the coupled CN^{-} orientation/Cd-displacement model used to calculate the X-ray diffuse scattering in the main text (Figure 4d).

SUPPLEMENTARY TABLES

Supplementary Table 1. Cell energies and effective CN^- interaction parameters determined using QC calculations for $Cd(CN)_2$. The labels 's' and 'd' denote single-network and interpenetrated-network configurations, as described in the text.

Calculation method	ΔE (kJ per mol Cd)				J_{eff} (K)	D (K)	Δ (K)
	C_3N/CN_3		C_4/N_4				
	s	d	s	d			
PBE-D3 CP2K	0.97	3.16	4.20	6.74	191	93	12 800
optPBE-vdW CP2K	1.10	4.15	3.60	7.44	160	144	10 500
PBE CP2K	1.07	1.17	4.17	4.50	188	15	13 000
BLYP CP2K	1.68	1.06	3.65	4.23	154	37	10 800
rVV10 CP2K	1.19	1.08	3.79	4.46	168	34	10 500
BLYP-D3 CP2K	1.02	14.43	4.02	18.14	181	500	-
PBE0 CP2K	1.09	1.20	4.19	4.52	188	15	-
PBE CASTEP	0.96	1.00	3.66	3.93	164	13	-
SCAN VASP	0.95	0.93	3.27	3.68	146	20	-

Supplementary Table 2. Cell energies and effective CN^- interaction parameters determined using QC calculations for $Zn(CN)_2$. The labels 's' and 'd' denote single-network and interpenetrated-network configurations, as described in the text.

Calculation method	ΔE (kJ per mol Zn)				J_{eff} (K)	D (K)	Δ (K)
	C_3N/CN_3		C_4/N_4				
	s	d	s	d			
PBE-D3 CP2K	0.26	0.28	0.95	1.05	43	6	14 400

Supplementary Table 3. Crystallographic refinement details for $Cd(CN)_2$ measured on cooling between 140 < T < 300 K. Space group $Pn\bar{3}m$. The structure is defined by two atomic positions: Cd (¼,¼,¼), Wyckoff position 4*b* and C/N (*x*,*x*,*x*), Wyckoff position 8*e*, occupancy $C_{0.5}N_{0.5}$.

T (K)	a (Å)	x	B_{iso}(Cd) (Å^2)	B_{iso}(C/N) (Å^2)	R_{wp} (%)
300	6.30760(18)	0.05376(17)	13.5(2)	9.62(12)	2.769
290	6.30714(18)	0.05363(17)	13.2(2)	9.60(12)	2.837
280	6.30663(18)	0.05376(17)	13.3(2)	9.41(12)	2.877
270	6.30851(18)	0.05392(17)	13.4(2)	9.60(12)	2.878
260	6.30976(19)	0.05374(17)	13.2(2)	9.43(12)	2.881
250	6.31074(18)	0.05409(17)	13.3(2)	9.17(12)	2.905
240	6.31261(18)	0.05414(17)	13.4(2)	9.23(12)	2.908
230	6.31310(19)	0.05443(17)	13.4(2)	9.11(12)	2.930
220	6.31522(19)	0.05425(16)	13.5(2)	8.95(11)	2.958
210	6.31600(19)	0.05430(16)	13.3(2)	9.03(11)	2.956
200	6.31779(19)	0.05428(16)	13.0(2)	8.74(11)	2.953
190	6.31858(19)	0.05427(16)	13.6(2)	8.76(11)	2.988
180	6.32067(18)	0.05469(16)	13.5(2)	8.69(11)	2.973
170	6.32143(19)	0.05461(16)	13.4(2)	8.80(11)	3.076
160	6.32342(19)	0.05456(16)	13.9(2)	8.91(11)	3.151
150	6.32492(19)	0.05461(17)	13.8(3)	8.80(12)	3.295
140	6.3290(2)	0.05426(18)	14.6(3)	9.10(13)	2.769

Supplementary Table 4. Crystallographic refinement details for $Cd(CN)_2$ between 10 < T < 130 K including the tetragonal lattice parameters and the occupancy of carbon on the C site, n. The carbon and nitrogen occupancies on the C and N sites are constrained such that the total occupancy sums to unity on each and that their values are each other's inverse: C_nN_{1-n}(C site) = N_nC_{1-n}(N site).

T (K)	a (Å)	c (Å)	V (Å^3)	n	R_{wp} (%)
140	8.95(2)	12.664(7)	1014.1(8)	0.57(4)	2.769
130	8.9800(11)	12.644(3)	1019.6(4)	0.55(3)	2.837
120	9.0047(10)	12.628(3)	1023.9(3)	0.58(3)	2.877
110	9.0235(8)	12.615(2)	1027.2(2)	0.67(3)	2.878
100	9.0373(7)	12.6084(17)	1029.8(2)	0.66(3)	2.881
90	9.0481(6)	12.6034(14)	1031.81(18)	0.68(2)	2.905
80	9.0591(6)	12.5989(13)	1033.96(17)	0.72(2)	2.908
70	9.0656(5)	12.5952(12)	1035.15(15)	0.73(2)	2.930
60	9.0727(5)	12.5940(11)	1036.67(15)	0.70(2)	2.958
50	9.0776(5)	12.5930(11)	1037.70(13)	0.696(19)	2.956
40	9.0842(5)	12.5865(11)	1038.68(13)	0.712(19)	2.953
30	9.0891(4)	12.5853(10)	1039.70(13)	0.691(18)	2.988
20	9.0917(4)	12.5868(9)	1040.42(12)	0.716(17)	2.973
10	9.0923(4)	12.5867(9)	1040.54(12)	0.714(17)	2.957

Supplementary Table 5. Crystallographic refinement details for $Cd(CN)_2$ between $10 < T < 130$ K including atomic positions. Space group $I4_1/amd$. The structure is defined by three atomic positions: Cd (0,0,*z*), Wyckoff position 8*e*; C (0,*y*,*z*), Wyckoff position 16*h;* and N (*x*,0,*z*), Wyckoff position 16*h*. The isotropic thermal displacement parameter B_{iso} was constrained to be the same for C and N.

T (K)	*z*(Cd)	*x*(N)	*z*(N)	*y*(C)	*z*(C)	B_{iso}(Cd) (Å^2)	B_{iso}(C/N) (Å^2)
130	0.7196(8)	0.1991(8)	0.6255(5)	0.3010(10)	0.4283(5)	7.1(2)	3.76(9)
120	0.7168(7)	0.1973(7)	0.6305(5)	0.3045(9)	0.4332(4)	5.8(2)	2.91(7)
110	0.7148(7)	0.1964(6)	0.6337(4)	0.3040(8)	0.4361(4)	5.00(18)	2.55(6)
100	0.7134(7)	0.1955(6)	0.6362(4)	0.3024(7)	0.4386(4)	4.61(16)	2.35(6)
90	0.7120(7)	0.1950(5)	0.6384(3)	0.3023(7)	0.4405(3)	4.56(15)	2.24(4)
80	0.7098(7)	0.1948(4)	0.6395(3)	0.3018(6)	0.4427(3)	4.48(14)	2.11(5)
70	0.7087(6)	0.1950(4)	0.6414(3)	0.3017(6)	0.4441(3)	4.27(13)	1.98(4)
60	0.7076(6)	0.1942(4)	0.6419(3)	0.3008(5)	0.4448(3)	4.27(13)	1.88(4)
50	0.7062(6)	0.1952(4)	0.6433(3)	0.3018(5)	0.4459(3)	4.18(12)	1.82(4)
40	0.7054(6)	0.1953(4)	0.6441(3)	0.3017(5)	0.4474(2)	4.23(12)	1.65(4)
30	0.7049(6)	0.1955(4)	0.6452(3)	0.3022(5)	0.4482(2)	3.96(11)	1.54(3)
20	0.7030(6)	0.1954(3)	0.6450(2)	0.3021(4)	0.4484(2)	3.71(10)	1.46(3)
10	0.7027(6)	0.1954(3)	0.6452(2)	0.3020(4)	0.4486(2)	3.70(10)	1.46(3)

Supplementary Table 6. Fractional populations of the $CdC_{4-x}N_x$ configurations determined by variable-temperature solid-state NMR measurements (RT = room temperature).

T	$P(C_2N_2)$ (%)	$P(C_3N) = P(CN_3)$ (%)	$P(C_4) = P(N_4)$ (%)
−100 °C	55.1	22.2	0.3
25 °C	50.2	23.2	1.7
100 °C	52.6	22.1	1.6
RT (fast MAS)	51.5	22.8	1.4

Supplementary Table 7. Exchange rates k_{ab} between $CdC_{4-x}N_x$ configurations where $b = x$ before and $a = x$ after the exchange. The values correspond to direct jumps between both states without crossing a stable intermediate.

T (°C)	k_{12} (s^{-1})	k_{32} (s^{-1})	k_{13} (s^{-1})
85	44(8)	43(6)	13(11)
67	11(2)	10(2)	0(2)
62	9(1)	8(2)	0(2)

SUPPLEMENTARY METHODS

Neutron diffraction study of kinetic trapping of CN order

An additional neutron diffraction experiment was carried out using the POLARIS instrument at the ISIS facility and using the same $^{114}Cd(CN)_2$ sample as for the primary measurements. The sample was cooled from room temperature to 30 K and held at 30 K for a period of one month before heating to 125 K, then re-cooled to 30 K. The spontaneous strain and cyanide order parameters for these cycles are shown in Supplementary Figure 2.

Rietveld analysis

Rietveld analysis of the variable temperature neutron powder diffraction data was carried out using the TOPAS software[1]. Data from the three highest–angle detector banks on POLARIS were used for structure refinement as no additional reflections with d > 7 Å were present in the two banks at lowest scattering angles. The instrumental time-of-flight zero offsets (t_0, t_1, t_2) were determined by refining against a Si SRM 640b standard. The value of t_2 was refined against the dataset measured for $Cd(CN)_2$ at 300 K and subsequently fixed for all other temperatures down to 10 K. Structural parameters that were refined included: the lattice parameters, atomic positions, and atomic displacements parameters (C/N were constrained to have the same atomic displacement parameter). Lorentzian and Gaussian strain terms were also refined to account for sample-related peak broadening. For temperatures $\leq$ 130 K, the choice of space-group $I4_1/amd$ allows for distinct C and N positions. Their occupancies were initialised to be $C_{0.5}N_{0.5}$ (and constrained to sum to unity) at 130 K and subsequently allowed to refine for all temperatures down to 10 K. The key crystallographic results of these refinements are given in Supplementary Tables 3–5.

Neutron PDF analysis

The fractional populations of Cd environments were estimated from the neutron pair distribution function $G(r)$ by a constrained fitting to the peaks between 3.1 and 3.8 Å. The peaks in this region correspond to pairwise correlations across Cd–(C)–N/Cd–(N)–C and C–(Cd)–C/C–(Cd)–N/N–(Cd)–N triplets. The positions and intensities of these peaks were found to be the most sensitive to the local coordination environment around Cd [Supplementary Figure 11]. At lower values of r, the peaks correspond to C–N, Cd–C and Cd–N bonds, which are invariant with respect to CN^- flips. At higher values of r, the effects of inter-framework (as well as intra-framework) correlations become important and the interpretation becomes much more complex. Detailed analysis of the pair-distribution function is reserved for future work.

In order to extract from the variation in $G(r)$ the local CdC_xN_{1-x} coordination-environment populations using the least number of fitting parameters, we carried out the following calculations. For each of the interpenetrated-network $Cd(CN)_2$ configurations generated in our QC calculations—one with CdC_2N_2 environments ($\gamma = 0$), one with $CdCN_3$/$CdNC_3$ environments ($\gamma = \frac{2}{3}$), and the third with CdC_4/CdN_4 environments ($\gamma = \frac{8}{3}$)—we determined the corresponding neutron PDFs using the software PDFGUI[2]. The differences between the experimental $G(r)$ function at 300 K and that measured at lower temperatures were then

fitted using a linear combination of the calculated $G(r)(CdC_2N_2)$ and $G(r)(CdC_3N/CdCN_3)$ functions. The fraction of CdC_4/CdN_4 environments was assumed to be negligible in order to simplify the model such that only one parameter—*viz.* the ratio of CdC_2N_2 to $CdC_3N/CdCN_3$—was refined against the experimental $G(r)$ function. This ratio was determined by least-squares minimisation of the squared-difference between the calculated and experimental $G(r)$ functions, as implemented in the OriginPro 2017 software. Essentially what this analysis does is to interpret the increase in scattering intensity at $r \simeq 3.6$ Å observed on cooling in terms of an increasingly large fraction of CdC_2N_2 coordination environments.

Population analysis from NMR measurements

Based on the quantitative 1D spectra measured at different temperatures, the temperature-dependent fractional populations of the $CdC_{4-x}N_x$ species were determined as follows. Assignment of the isotropic signal was carried out based on the assignment in Supplementary Reference 3. The intensities corresponding to the individual Cd environments were calculated as the sum of the integrals of the isotropic signal and two sidebands on each side. The relative positions of these sidebands were set according to $\nu = \nu_{\mathrm{iso}} \pm n\nu_{\mathrm{rot}}$ with ν_{rot} the spinning speed and $n = \pm 1, \pm 2$.

For CdC_4 and CdN_4 resonances only isotropic signals were refined due to their poor signal-to-noise ratio. One common Gauss-Lorentz ratio was refined for all signals, and the peak width was refined individually for each $CdC_{4-x}N_x$ species.

Simple mass-balance considerations constrain the populations P of the various species: $4P(\mathrm{C_4}) + 3P(\mathrm{C_3N}) + 2P(\mathrm{C_2N_2}) + P(\mathrm{CN_3}) = 4P(\mathrm{N_4}) + 3P(\mathrm{CN_3}) + 2P(\mathrm{C_2N_2}) + P(\mathrm{C_3N})$, which simplifies to $2P(\mathrm{C_4}) + P(\mathrm{C_3N}) = 2P(\mathrm{N_4}) + P(\mathrm{CN_3})$. Due to the poor signal-to-noise ratio for the CdC_4 and CdN_4 resonances, the parameter set was further reduced by the additional constraints $P(\mathrm{C_4}) = P(\mathrm{N_4})$ and $P(\mathrm{C_3N}) = P(\mathrm{CN_3})$. Whereas the summed intensity of a Cd environment is restrained by the conditions above, the relative shares of isotropic shift and sidebands contributing to it were allowed to vary freely.

The simulated spectra constructed from the constrained set of signals were refined by least-squares minimisation compared to the experimental NMR spectra at 100, 25, and –100 °C. For the NMR spectrum measured with a spinning speed of 20 kHz [Supplementary Figure 5], refinements were performed the same way, but only one sideband at each side ($n = \pm 1$) was fitted for CdC_2N_2, CdC_3N and $CdCN_3$. The resulting fractional populations are reported in Supplementary Table 6.

CN dynamics from NMR exchange spectroscopy

The rate of a single CN^- flip is equivalent to the exchange rate between CdC_2N_2 and CdC_3N, or between CdC_2N_2 and $CdCN_3$. Accordingly, a transformation between CdC_3N and $CdCN_3$ configurations corresponds to two flips of different CN^- molecules. Since the $CdC_{4-x}N_x$ species are uniquely identified by their isotropic shift, their dynamics and thus those of the CN^- ion can be investigated by exchange NMR spectroscopy (EXSY). The time-dependent development in EXSY measurements can be described by employing the rate matrix

approach[4–6]: the signal intensities observed in the spectra are proportional to conditional probabilities p_{ab} for all possible internuclear exchange paths, which are connected with the corresponding exchange rates in the master equation:

$$\dot{P} = KP, \qquad (1)$$

where P is the matrix of conditional probabilities and K the kinetic matrix. The probability matrix elements p_{ab} represent the likelihood to find magnetisation on nucleus a after the mixing interval that was located on nucleus b at the beginning. Correspondingly, the kinetic matrix elements k_{ab} describe the time-independent exchange rates for a direct jump from b to a. Implicit in Supplementary Equation (1) is the time-invariance of the underlying experiment; *i.e.* that the conditional probabilities are solely determined by the previous step, whereas prior development is irrelevant—a Markov process. Detailed balance then requires

$$k_{ab}p_b^{\mathrm{eq}} = k_{ba}p_a^{\mathrm{eq}}, \qquad (2)$$
$$k_{aa} = -\sum_{b\neq a} k_{ab}, \qquad (3)$$

where p_s^{eq} is the population of the particular $CdC_{4-x}N_x$ species s at equilibrium.

Neglecting the CdC_4 and CdN_4 populations due to their low signal-to-noise ratio, we obtained from our quantitative fast MAS data

$$k_{21} = k_{23} = \frac{0.530}{0.235}k_{12} = \frac{0.530}{0.235}k_{32}, \qquad (4)$$
$$k_{13} = k_{31}, \qquad (5)$$

where the indices correspond to the value *x* in $CdC_{4-x}N_x$.

Supplementary Equation (1) solves to

$$P(\tau_{\mathrm{mix}}) = P_0 \exp(K\tau_{\mathrm{mix}}), \qquad (6)$$

where τ_{mix} is the mixing time (see Supplementary Figure 4) and the vector P_0 contains the populations p_s^0 of the configurations s at the beginning of the experiment. For selective excitation of a single resonance, P_0has only one non-zero element; *e.g.* $[010]^{\mathrm{T}}$ for selective excitation of the *x* = 2 environment (CdC_2N_2).

The evolution of the conditional-probability matrix $P(\tau_{\mathrm{mix}})$ as a function of mixing time is described by a matrix of buildup curves (Supplementary Figure 6), themselves derived from the deconvoluted EXSY spectra of each temperature (Supplementary Figures 7–9). This three-dimensional dataset was then fitted using the three independent variables in the kinetic matrix—namely, the rates k_{12}, k_{13}, and k_{32}. The corresponding values are given in Supplementary Table 7. Since the transfer from CdC_2N_2 to CdC_3N and from CdC_2N_2 to $CdCN_3$ effectively describe the same underlying process—a CN^-flip—an Arrhenius plot of both k_{12} and k_{32} (Supplementary Figure 10) was used to determine the CN flip activation energy of 73(5) kJ mol^{-1}. The values of the k_{13} jump rates are zero or very close to zero within the error margin.

The experimental considerations that have constrained the number and temperature-range of our EXSY measurements can be summarised as follows. These measurements are extremely time-consuming, especially as temperature is reduced. The lower bound of 60 °C arises from the long spin–lattice (T_1) relaxation time of ^{113}Cd in $Cd(CN)_2$, which is such that obtaining suitable statistics in the build-up curves for lower temperatures was clearly impractical. The probe itself is not designed to operate at temperatures above 85 °C. In addition, this temperature is also close to the limit of the measurement technique for this sample, since further increase in jump rates will lead to flips during the initial selective excitation and so prevent a meaningful result. Taken together this is why we have had to content ourselves with relatively few measurements within a narrow temperature window.

SUPPLEMENTARY DISCUSSION

Sensitivity of neutron diffraction to CN orientations

A key result presented in the main text is the emergence of CN^- orientational order within the low-temperature $I4_1/amd$ phase (Figure 2d), to which we claim neutron scattering is sensitive given the contrast in C and N scattering lengths (and, as established in other cyanide-containing frameworks, see *e.g.* Supplementary Reference 7). In Supplementary Figure 4 we show explicitly the difference in calculated neutron scattering patterns for one of our $Cd(CN)_2$ measurements before and after reversal of CN^- orientations. The key point is that the corresponding differences in reflection intensities are much larger than the uncertainty in the measured data, and hence our data are indeed sensitive to long-range CN^- order.

Additional quantum chemical calculations

Out of curiosity, and for completeness, we also carried out a series of parallel calculations to assess functional and van der Waals dispersion effects in CP2K using PBE[8], BLYP[9], optPBE-vdW[10], rVV10[11], and the hybrid functional, PBE0[12]. We also checked for consistency between different DFT codes. Optimisation was also performed in VASP[13–15] with the SCAN functional[16]. VASP uses the projector-augmented wave method (PAW)[17] to describe the interactions between the core electrons and valence electrons. CASTEP[18] and the PBE functional[8] were also used, with pseudopotentials created on the fly. In all cases, energy cut-offs, convergence criteria and *k*-point sampling were kept constant. The corresponding energies and derived parameters are listed in Supplementary Table 1. Note that we consider the PBE-D3 (CP2K) results to be the highest quality data set. Nevertheless, in general, the energies obtained are internally consistent between functionals with and without van der Waals dispersion, which indicates that long-range dispersion is not decisive and that short-range electrostatics control the energy landscape. The outlier in the data is BLYP-D3 which potentially indicates a parameterisation problem for this particular functional with the D3 method for approximating dispersion interactions.

We also calculated the QC energies for equivalent cells of $Zn(CN)_2$; the corresponding PBE-D3 (CP2K) results are given in Supplementary Table 2.

Finally, we also repeated our nudged elastic band calculations for each of the functionals PBE[8], PBE-D3[19], BLYP[9], optPBE-vdW[10], rVV10[11], and PBE0[12]. The corresponding values of Δ extracted from all data sets are given in Supplementary Tables 1 ($Cd(CN)_2$) and 2 ($Zn(CN)_2$).

Contribution of CN flips to thermal expansion

Since $Cd(CN)_2$ is a negative thermal expansion material, and since CN^- flips result in a reduction of the Cd…Cd distance, we sought to establish the extent to which those fluctuations of CN^- ions captured by our spin-ice model might contribute to the experimental NTE. In estimating this contribution, we first determine the temperature dependence of the expectation value of the parameter $S_{\parallel}$, which characterises the average projection of the CN^- pseudospins onto their local $\langle 111 \rangle$ axes. The central concept is that

the average Cd…Cd separation depends on this parameter: when $S_{\parallel} = 1$ the system is in the athermal limit and the Cd…Cd separation is given most closely by that observed in the (relaxed) *ab initio* configuration; when $S_{\parallel} = 0$ the system is at the transition-state for a CN^- flip, and the Cd…Cd separation is reduced that observed at the transition-state shown in Figure 3c of the main text. These values were $d(\mathrm{Cd} \ldots \mathrm{Cd})_0$ = 5.545 Å and $d(\mathrm{Cd} \ldots \mathrm{Cd})^{\ddagger}$ = 4.898 Å, respectively (Supplementary Figure 6). In order to determine $S_{\parallel}$, we take the single-ion energy calculated in our MC simulations and exploit the relationship $\langle S_{\parallel} \rangle = \sqrt{E_{\text{single-ion}}/\Delta}$. We then have $d(T)$ = 4.898 Å + [0.647 Å × $\langle S_{\parallel}(T) \rangle$], and finally the expected coefficient of thermal expansion $\alpha = \mathrm{d}d/d\mathrm{d}T$ calculated from the temperature derivative of *d*(*T*) calculated over the temperature range 120–600 K.

The values we determine are –6.4 MK^{-1} for $Cd(CN)_2$ and –4.42 MK^{-1} for $Zn(CN)_2$; the corresponding experimental values are –20.4(4) and –16.9(2) MK^{-1}, respectively. Note that we do not expect equivalence here, since the low-energy phonons responsible for NTE in both materials include CN^- translational modes, which are not captured by our pseudospin model[20–22]. Instead we note simply that the spin-ice CN^- fluctuations in both materials constitute ~30% of the observed NTE effect, and that the ordering of strengths of NTE effects for $Cd(CN)_2$ *vs* $Cd(CN)_2$ is as expected.

SUPPLEMENTARY REFERENCES

1. Coehlo, A. A. TOPAS-Academic, version 4.1 (Computer Software) (2007).

2. Farrow, C. L., Juhás, P., Liu, J. W., Bryndin, D., Božin, E. S., Bloch, J., Proffen, Th. & Billinge, S. J. L. PDFfit2 and PDFgui: computer programs for studying nanostructure in crystals. *J. Phys.: Cond. Mat.* **19**, 335219 (2007).

3. Nishikiori, S.-I., Ratcliffe, C. I. & Ripmeester, J. A. ^{113}Cd NMR studies of Hofmann-type clathrates and related compounds: Evidence for two room temperature orientational glasses. *Can. J. Chem.* **68**, 2270–2273 (1990).

4. Robyr, P., Tomaselli, M., Straka, J., Grobpisano, C., Suter, U. W., Meier, B. H. & Ernst, R. R. RF-driven and proton-driven NMR polarization transfer for investigating local order – An application to solid polymers. *Mol. Phys.* **84**, 995–1020 (1995).

5. Wittebort, R. J. & Szabo, A. Theory of NMR relaxation in macromolecules: Restricted diffusion and jump models for multiple internal rotations in amino acid side chains. *J. Chem. Phys.* **69**, 1722–1736 (1978).

6. Senker, J. Molecular dynamics of amide ions in potassium amide (KNH_2) studied with orientation-dependent deuterium spin lattice relaxation. *Solid State Nucl. Magn. Reson.* **26**, 22–35 (2004).

7. Goodwin, A. L., Calleja, M., Conterio, M. J., Dove, M. T., Evans, J. S. O., Keen, D. A., Peters, L. & Tucker, M. G. Colossal positive and negative thermal expansion in the framework material $Ag_3[Co(CN)_6]$. *Science* **319**, 794–797 (2008).

8. Perdew, J. P., Burke, K. & Ernzerhof, M. Generalized gradient approximation made simple. *Phys. Rev. Lett.* **77**, 3865–3868 (1997).

9. Becke, A. D. Density-functional exchange-energy approximation with correct asymptotic behaviour. *Phys. Rev. A* **38**, 3098–3100 (1988).

10. Klimeš, J., Bowler, D. R. & Michaelides, A. Van der Waals density functionals applied to solids. *Phys. Rev. B* **83**, 195131–195160 (2011).

11. Sabatini, R., Gorni, T. & de Gironcoli, S. Nonlocal van der Waals density functional made simple and efficient. *Phys. Rev. B* **87**, 041108 (2013).

12. Perdew, J. P., Ernzerhof, M. & Burke, K. Rationale for mixing exact exchange with density functional approximations. *J. Chem. Phys.* **105**, 9982–9985 (1996).

13. Kresse, G. & Hafner, J. Ab initio molecular dynamics for liquid metals. *Phys. Rev. B* **47**, 558–561 (1993).

14. Kresse, G. & Furthmüller, J. Efficiency of ab-initio total energy calculations for metals and semiconductors using a plane-wave basis set. *Comput. Mater. Sci.* **6**, 15–50, (1996).

15. Kresse, G. & Furthmüller, J. Efficient iterative schemes for ab initio total-energy calculations using a plane-wave basis set. *Phys. Rev. B* **54**, 11169–11186 (1996).

16. Mezei, P. D., Csonka, G. I. & Kállay, M. Simple modifications of the SCAN meta-generalized gradient approximation functional. *J. Chem. Theory Comput.* **14**, 2469–2479, (2018).

17. Blöchl, P. E. Projector augmented-wave method. *Phys. Rev. B* **50**, 17953–17979 (1994).

18. Clark, S. J., Segall, M. D., Pickard, C. J., Hasnip, P. J., Probert, M. J., Refson, K. & Payne, M. C. First principles methods using CASTEP. *Z. Kristallogr.* **220**, 567–570, (2005).

19. Grimme, S. A. J., Ehrlich, S. & Krieg, H. A consistent and accurate ab initio parameterization of density functional dispersion correction (DFT-D) for the 94 elements H-Pu. *J. Chem. Phys.* **132**, 154104–154118 (2010).

20. Goodwin, A. L. & Kepert, C. J. Negative thermal expansion and low-frequency modes in cyanide-bridged framework materials. *Phys. Rev. B* **71**, 140301 (2005).

21. Chapman, K. W., Chupas, P. J. & Kepert, C. J. Direct observation of a transverse vibrational mechanism for negative thermal expansion in $Zn(CN)_2$: An atomic pair distribution function analysis. *J. Am. Chem. Soc.* **127**, 15630–15636 (2005).

22. Fang, H., Dove, M. T., Rimmer, L. H. N. & Misquitta, A. J. Simulation study of pressure and temperature dependence of the negative thermal expansion in $Zn(CN)_2$. *Phys. Rev. B* **88**, 104306 (2013).
